# Dr. Kendall

**Published:** 1901-12

**Authors:** 


### Obituary.

---

### Dr. KENDALL.

From the *Western Morning News* of October 25th, 1901, we obtain the following particulars relating to the death of Dr. Kendall at Helston, at the age of 36:—"Very few but the most intimate friends of the family were aware that he had been indisposed for a day or two. He was away for several hours on Monday night attending a serious case, and probably returning home in the early hours of the morning caught cold, which settled in his throat, resulting in laryngitis. He was attended by Drs. Henry and Wearne; and up to a short time before his death it was not thought that his condition was likely to have a fatal termination. The deceased gentleman, born in India, was the son of Surgeon-Major Kendall, now retired from the army, and living at Upper Norwood, London; but the family home had been at Tenby, South Wales, since leaving Clifton some ten or twelve years ago. In 1895 Dr. Kendall took over the practice of Dr. C. F. Seville on the latter leaving Helston, and soon became known as a skilful practitioner. Before coming to Helston he studied for some years at the Royal Infirmary, Bristol. At the November Municipal Election, 1898, Dr. Kendall was returned at the head of the poll, his term of office expiring at the end of the present month. Only a day or two since, owing to his numerous professional engagements, he declined to be again nominated for the Council. Two years and a half ago he married Miss Isobel Teresa Hamilton, second daughter of Mr. E. P. Kendall, J.P., Bank Manager, Helston, of which there has been issue a boy seventeen months old and a girl one month. The deceased was Medical Officer and Vaccinator for Wendron District. A few weeks since he resigned his position; but at the unanimous request of the Guardians, many of whom spoke in the highest terms of his skill, and more especially of his unfailing kindness and sympathy with the poor, he withdrew his resignation."

His old fellow-students and friends in Bristol will regret that the venerable-looking and genial Bernard Kendall should have succumbed so early in life, and will sympathise deeply with his young widow in her bereavement.

